# The role of health determinants in the influenza vaccination uptake among older adults (65+): a scope review

**DOI:** 10.1007/s40520-021-01793-3

**Published:** 2021-02-15

**Authors:** Regina Roller-Wirnsberger, Sonja Lindner, Lea Kolosovski, Elisabeth Platzer, Peter Dovjak, Holger Flick, Chariklia Tziraki, Maddalena Illario

**Affiliations:** 1 Department of Internal Medicine/Geriatrics, Medical University of Graz, Graz, Austria; 2 Division of Nephrology, Department of Internal Medicine, Medical University of Graz, Graz, Austria; 3 Department of Acute Geriatrics, Salzkammergut Klinikum Gmunden, Gmunden, Austria; 4 Division of Pneumology, Department of Internal Medicine, Medical University of Graz, Graz, Austria; 5 Clinic of Social and Family Medicine, School of Medicine, University of Crete, Crete, Greece; 6 Federico II Department of Public Health, Naples, Italy

**Keywords:** Influenza vaccination, Health determinants, Older adults, Vaccination uptake, Vaccination hesitancy

## Abstract

**Background:**

Although the burden of influenza infection is the highest in older adults, vaccination coverage remains low, despite this age group being more vulnerable than others.

**Aims:**

Given the current pandemic of SARS-CoV-2, it was the aim of this scope review to update knowledge on factors affecting seasonal influenza vaccine uptake among older adults to strengthen prevention approaches in the context of an overall burden of infectious diseases.

**Methods:**

We searched bibliographic databases from 2012 to 2019. All studies reviewed one or more social determinant of health listed by WHO, or factors affecting the decision-making process whether to accept influenza vaccine or not.

**Results:**

Overall, 44 studies were included, 41 determinants were extracted and summarized into six categories. Older age and constitutional factors including multiple chronic diseases as well as preventive lifestyle and frequent routine healthcare utilization positively affected vaccination uptake (VU). Living and working conditions are also researched determinants of influenza vaccine uptake. A small number of studies explored the role of social inclusion and system-based interventions.

**Discussion and conclusions:**

This scope review provides a comprehensive overview on factors affecting seasonal influenza vaccination uptake among older citizens. The review also clearly shows gaps for evidence on system-based level or political strategies to improve vaccination uptake.

**Supplementary Information:**

The online version contains supplementary material available at 10.1007/s40520-021-01793-3.

## Introduction

Public health interventions and the understanding of health as a social continuum over the whole life span requires complex interactions with care systems to facilitate health for as many citizens as possible [1]. Social determinants of health as developed by the World Health Organization (WHO) in 2010, provide a conceptual framework particularly useful for global policy making within this complex networks and interactions [2], as they play a critical role in disease occurrence, distribution and consequences. In its conceptual framework on the social determinants of health, three key components are addressed: the socio-political context, including governance, macroeconomic policy, social policies, public policy, culture and societal values as well as epidemiological conditions; structural determinants, including income, education, occupation, social class, gender, and race/ethnicity; and intermediary determinants, including material circumstances, social-environmental or psychosocial circumstances, behavioural and biological factors and the health system [2].

Given the current pressure on health care systems during the SARS-CoV-2 pandemic, which affects societies worldwide with a focus on adverse outcomes especially for older and vulnerable groups [3], any preventive measures to avoid infectious diseases and especially exploit the benefits of the vaccinations available to protect older people from suffering have come into focus. In this context, the World Health Organization (WHO) recommends seasonal influenza vaccination for older people on a yearly basis, targeting a 75% rate of vaccination for all countries [4]. Despite this recommendation, the intended coverage is not reached in many countries, especially in older adults, challenging health care systems in terms of hospitalization and mortality rates [5].

Reasons for limited vaccination uptake (VU) and hesitancy (VH) have been studied in various studies. A review by Nagata JM and colleagues tried to summarize data on vaccination uptake for seasonal influenza among adults aged 65 years and older [6]. However, since this publication, the needs of health care systems as well as citizens have been strikingly affected by SARS-CoV-2 pandemic, building impact on health and social behaviour, economic capacities as well as health beliefs, all of which have been shown to influence evidence-informed political decision-making [7]. It is, therefore, the aim of this scope review to systematically collect and analyse main determinants influencing seasonal influenza vaccination uptake of adults ≥ 65 years based on a systematic literature research in a complementary manner, to allow evidence-based decision-making for many stakeholders across the globe.

## Materials and methods

The review presented in this publication was conducted according to PRISMA guidelines [8]. Given the heterogeneous nature of results, the review is presented as scope review.

### Data resources

Relevant studies published between 1st January 2012 and 24th October 2019 in the English language were identified using PubMed, Cochrane library, CINAHL, Medline and Embase databases. Search strategy was applied by using the following search terms: “influenza” AND “vaccination” AND “older adults” AND “public health” AND”barrier” or refus* or strateg* or predict* or “health behaviour” or “health behavior”. If required, the medical subject headings were adapted to the specific database options with synonyms of the medical subject headings. Further search via greylit.org and reference tracking was performed to identify additional studies.

### Inclusion criteria

To broaden the evidence base for this review, no restrictions on study design were made. To be included, articles had to meet all of the following criteria: (1) Randomized controlled trials or non-randomized controlled trials, cross-sectional studies, cohort studies or reviews; (2) addresses factors–health determinants influencing influenza vaccination uptake and/or refusal; (3) included people aged 65 years or older; and/or (4) health and social workforce older than 18 years; and/or (5) care planners or those defined as responsible persons.

### Outcomes of interest and screening process

The primary focus of interest was health determinants as defined by the World Health Organization (WHO) [2] and their impact on vaccination uptake in a cohort of citizens older than 65 years. Each co-author contributed independently as a reviewer, based upon his/her experience in public health. Title- and abstract screening to exclude non-relevant articles was undertaken by one reviewer (L.K.). Subsequent full-text screening was completed by four independent reviewers (L.K., E.P., R.R.-W., S.L.) and non-eligible articles were excluded with justification. Selected publications were clustered according to the health determinants model of WHO by six reviewers (R.R.-W, M.I., C.T., P.D., H.F., S.L.) [2].

### Data synthesis and analysis according to health determinants

Meta-analysis was not performed due to the expected heterogeneity of the interventions. Relevant outcome data from the included studies were used to cluster the described factors that influence influenza vaccination uptake in older adults in the selected publications, according to social health determinants. Data were summarized at different levels: structural and intermediate determinants, all further clustered into policy and governance, provider and health care and patient level.

## Results

**Fig. 1:**
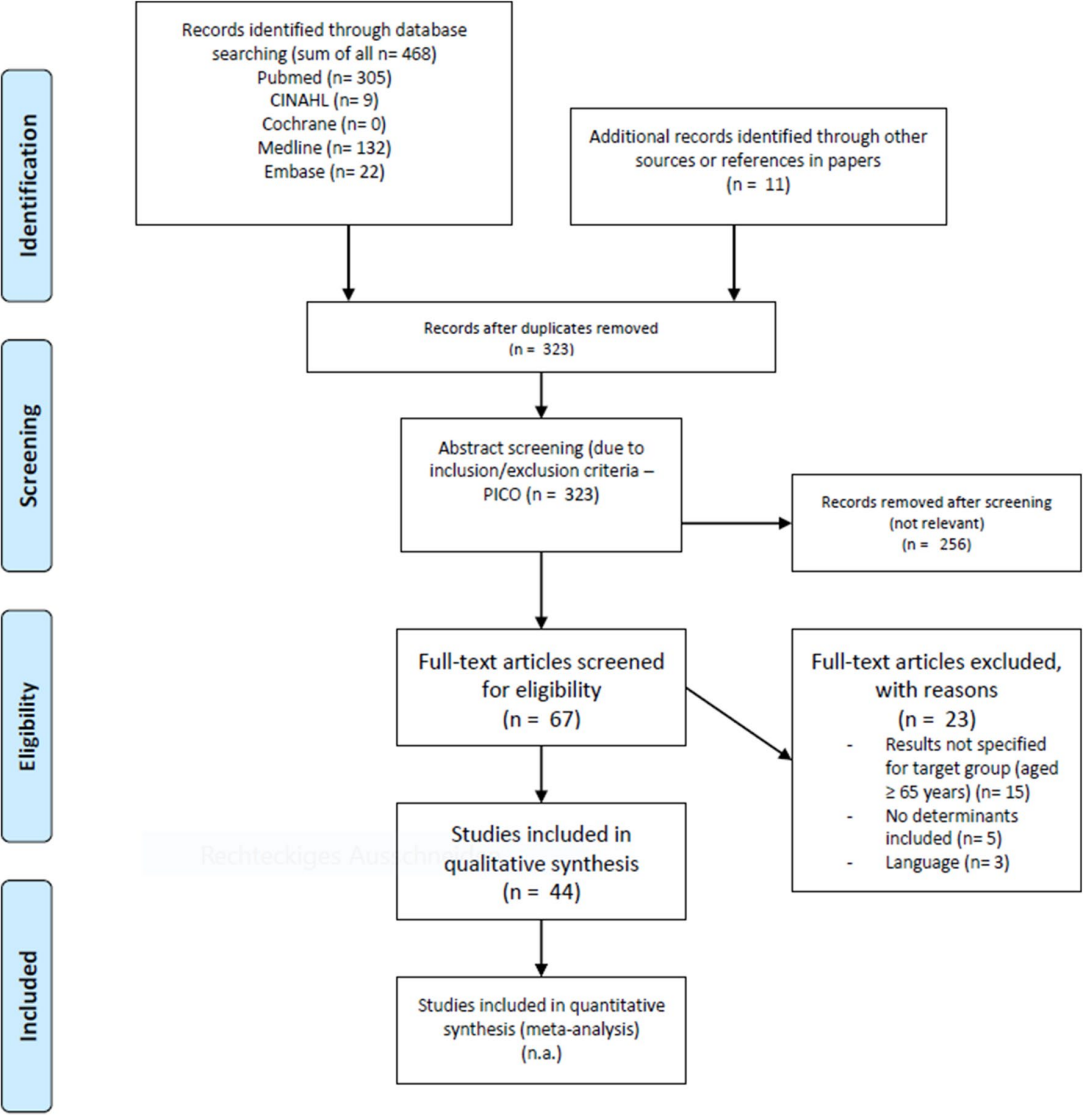
Prisma 2009 flow diagram. The flowchart illustrates the search strategy applied to answer the research question outlined. In total 479 studies were identified during the systematic data search (468 in scientific literature, 11 additional publications by hands-on search). Following qualitative evaluation and screening full text, 44 studies fulfilled predefined inclusion criteria of the publication and were further analysed in the review process

A total of 468 relevant citations were identified through a search strategy. Eleven additional studies were detected by a hands-on search. After identification of duplicate citations (*n* = 156 articles removed), title-/ abstract screening (*n* = 323 articles) and full-text screening (*n* = 67 articles), 44 studies met the inclusion criteria. The PRISMA diagram illustrates the selection process of the studies and shows reasons for exclusion (Fig. 1).

### Study characteristics

The study designs are divided into 34 cross-sectional studies [9–42] of which 13 studies were developed by means of a secondary analysis of already existing data [11, 12, 14, 15, 19–21, 28–30, 38, 39, 41] and two cross-sectional studies following a qualitative study design [26, 32], four randomized controlled trials [43–46], two systematic reviews [47, 48], two cohort studies [49, 50] and two theoretical reviews [51, 52]. Sample size ranged from 37 [32] to 13.106.163 [27] participants.

Methods used to evaluate vaccination uptake in the cross-sectional and cohort studies and in the systematic reviews were:

- questionnaires and surveys [13–15, 19, 21, 22, 25, 28, 34–36];
- telephone interviews [20, 23, 27, 29, 30, 33, 39, 41];
- data retrieved from national/regional authorities and health care institutions [12, 38, 40, 49, 50];
- data retrieved from medical records or vaccination registries [9, 10, 31, 37];
- combined methods [11, 17, 18, 24];
- focus group discussions [26, 32];
- face-to-face interviews [42];
- database search [47, 48].

One study did not indicate the detailed survey method used [16], yet was included because of its high relevance to the research topic.

The number of participants older than 65 years of study samples ranges from 11 to 100% of all initially included study participants (*n* = 19.604.711) from across the globe in countries from four continents and in following care settings: Community-dwelling or non-institutionalized citizens [9, 15, 21, 23, 27–31, 33, 36, 39, 42, 48, 50], nursing homes [12, 38], combined settings [32, 37], outpatient clinics [44], hospitals [18, 22], primary care centres/clinics or practices [24, 26, 43, 45], home-based primary care settings [10] and databases such as the Medicare registry, national vaccine industry or settings of health services/insurance authorities [13, 40, 46]. In 12 studies, details about the care setting of the participants were not indicated [11, 14, 16, 17, 19, 20, 25, 34, 35, 41, 47, 49].

Target groups for evaluation of factors influencing vaccination uptake at time of inclusion of the studies mentioned were adults from 65 years and older [9, 21, 25, 27, 28, 31, 33, 35, 41, 42, 50] (*n* = 13.884.163), persons at risk or with a certain (medical) condition over 65 years or with a sub-analysis of subjects older than 65 years [11, 17, 34, 45, 49, 51, 52] (*n* = 452.901), adults aged ≥ 65 years in a care setting [10, 18, 22, 26, 37, 44] (*n* = 390.102), healthy adults aged ≥ 18 years with a sub-analysis of subjects older than 65 years [20, 29, 30, 39] (*n* = 559.966), community-dwelling adults aged 60 years and older [36, 47, 48] (*n* = 1.056.678), nursing home residents [12, 32, 38] (*n* = 2.682.324), health care professionals providing care for patients older than 65 years [24] (*n* = 2.535), Medicare beneficiaries and patients older than 65 years enrolled in a national health insurance program [40, 46] (*n* = 364.944), combined target groups [23, 43] (*n* = 49.038), other related authorities [13] (*n* = 16) and adults of other age groups with a sub-analysis of subjects older than 65 years [14–16, 19] (*n* = 162.044). Further information on the baseline characteristics of the 44 studies included in this scope review can be drawn from Supplementary Table 1 (supplementary material).

### Clustering factors influencing influenza vaccination uptake according to WHO determinants of health

Reviewers (R.R.-W, M.I., C.T., P.D., H.F., S.L.) were asked to cluster factors described to influence influenza vaccination uptake in the selected publications according to WHO social health determinants, such as income, education, occupation, social class, gender and race/ethnicity [2]. This process resulted in defining 41 determinants that affect influenza vaccination uptake in adults ≥ 65 years according to this review. The factors found to affect VU and VH were summarized at different levels: structural and intermediate determinants, all further clustered into policy and governance, provider and health care and patient level. Determinants most analysed in connection with seasonal influenza vaccination are: age (*n* = 32 articles), gender (*n* = 30 articles), healthcare utilization or accessibility (n = 23 articles), education (*n* = 19 articles), income/socioeconomic status (*n* = 17 articles) and types of chronic diseases (*n* = 16 articles). Factors mostly lacking evidence for determining influenza vaccination behaviour were attitudes and behaviour of physician providing care (*n* = 3 articles), recommendations released by governmental bodies (*n* = 3 articles), level of care (*n* = 3 articles), dietary patterns, social networks and deprivation (each *n* = 2 articles), self-care (*n* = 1 article) and self-reported reasons, such as “no time” (*n* = 3 articles), “forgot” (*n* = 1 article), allergic reactions (*n* = 1 article) or “didn’t want it” (*n* = 2 articles). The remaining factors relate to personal experiences of the citizen, varying from the influence of family/friends (*n* = 4 articles) to household arrangements/children, previous vaccinations, and other health parameter (each *n* = 14 articles). The final workup of information according to presence in literature included into this review can be seen in Supplementary Table 2 (Supplementary material).

### Determinants and Ecosystem of factors affecting uptake of influenza vaccination globally in adults older than 65 years

Building on the clustering work presented in the previous section, we aligned information collected from the publications listed with social health determinants as outlined by WHO in 2010 [2]. Determinants clustered by their likelihood to increase or decrease VU for each article analysed can be seen in Supplementary Table 3.

**Fig. 2:**
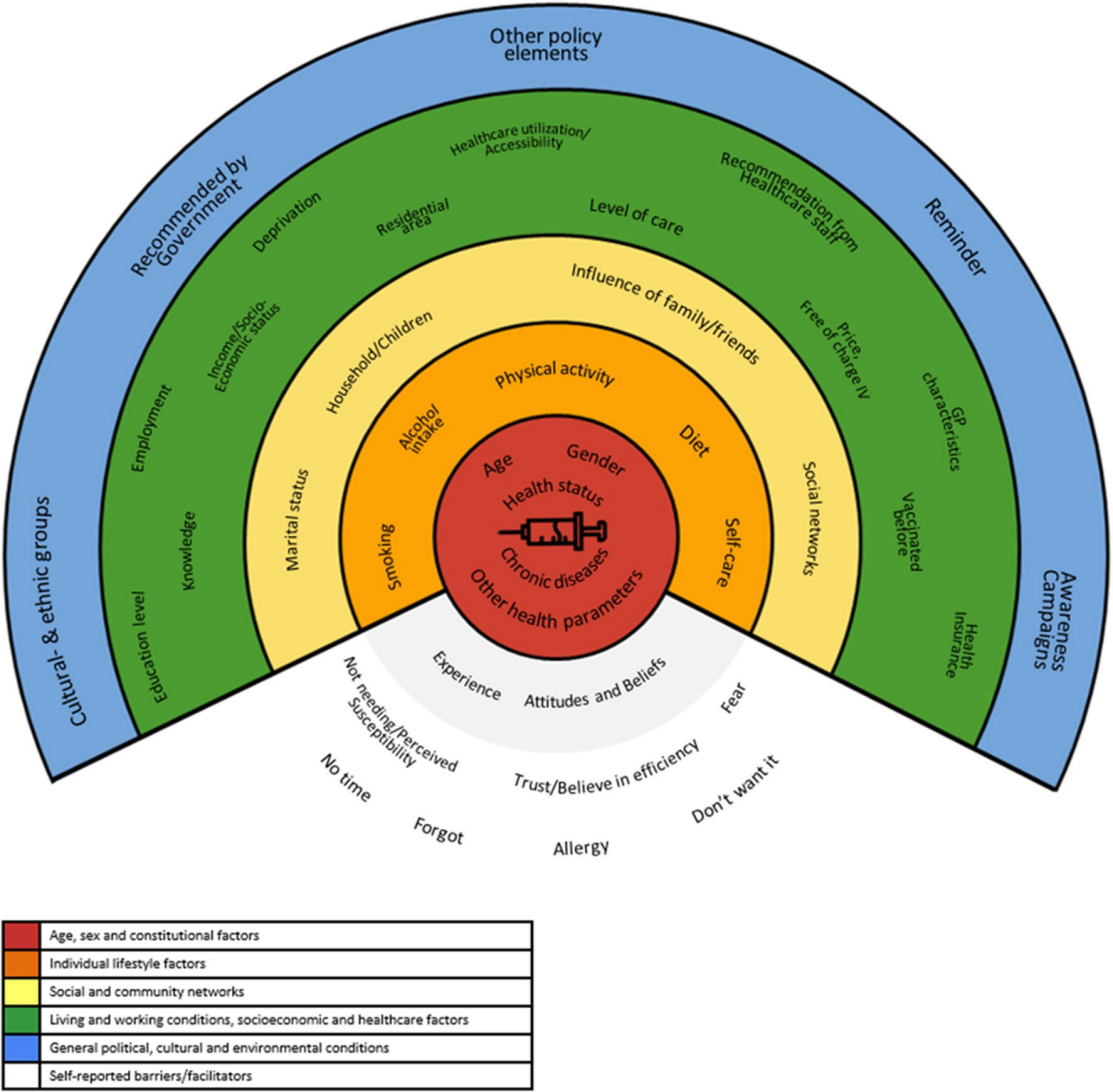
Ecosystem of factors affecting influenza vaccination behaviour. This shows the model of social health determinants adapted for the results obtained in this scope review on factors affecting vaccination uptake for seasonal influenza among citizens older than 65 years. The framework does not reflect numbers of publications found or numbers of participants included in the respective studies. It just gives an overall view on factors described for vaccination uptake and hesitancy currently described in the literature

We found sizeable evidence highlighting a role for several factors at the individual level, such as increasing age [11, 12, 20, 28, 34, 36, 39] and decreasing individual health status. The latter included declining functional status or having chronic diseases, comorbidities or disabilities [9–12, 14, 19, 20, 22, 25, 27–29, 33, 34, 37–39, 42], that supported VU among older people. Results related to gender were divergent, as some studies reported higher VU in females [12, 19, 33, 34, 42, 43] and others presented higher VU rates in males [9, 10, 21, 31, 40]. Besides those epidemiological and health parameters, health beliefs and experiences with recent vaccinations seem to impact VU for seasonal influenza vaccination [9, 15, 17, 18, 23, 26, 32, 33, 38, 41–43, 47, 51]. Not surprisingly, older citizens with positive attitudes towards VU also reported having other vaccinations such as pneumococcal vaccination [18]. Furthermore, lifestyle factors as smoking, low physical activity levels, inadequate diet and alcohol consumption seem to be negatively associated with VU [11, 14, 19, 28, 39]. Some studies point to the direction that a higher educational level or higher socioeconomic status may support VU in the elderly [20, 29, 39–41, 50]. Strong evidence was found in the field of healthcare utilization, showing that older citizens with more GP visits, health examinations or screenings and medical check-ups are more likely to receive influenza vaccination [9, 11, 14, 16–18, 26, 28–31, 34, 41]. In addition, interventions such as reminders, patient information/education or recommendations by health professionals seem to positively affect VU [22, 32, 33, 44–46, 48, 51, 52]. Moreover, Godoy et al. [24] highlighted that patients whose physicians were vaccinated had a higher VU than those whose physicians were not. An important result gathered in the review is the impact of social inclusion into family or informal social networks, which has been shown to positively affect VU [21, 22, 26, 33, 50]. Only a few publications were found reporting results of interventions on system level, one study found out that countries with good monitoring systems regarding VU rates exhibit higher vaccination coverage on average [13]. Additional policy elements also have the potential to increase VU rates [11, 13]. Figure 2 summarizes the single elements influencing seasonal influenza vaccination uptake among older citizens detected during our search in an ecosystem.

## Discussion

Influenza causes 400,000 influenza-associated deaths worldwide every year, and in Europe influenza has the highest burden of communicable diseases, followed by tuberculosis, human immunodeficiency virus infection and invasive pneumococcal disease [53–55].

This has important implications for the public health measures that can be implemented to respond to the virus.

Vaccination against seasonal influenza has proven effective, also reducing infection-associated death rates and is a highly recommended action during the SARS-CoV-2 pandemic [56, 57]. Despite evidence on efficacy being available, the effectiveness of vaccination is still lacking due to influenza vaccination hesitancy (VH), especially among older citizens [58]. VH is a complex behavioural phenomenon that may be influenced by a wide range of factors [59]. To achieve not only individual and community high vaccine-confidence, but also vaccine demand, context, population and vaccine-specific strategies need to be developed.

Our efforts were aimed at systematically collecting and analysing the main determinants influencing seasonal influenza vaccination uptake of adults ≥ 65 years in a complementary manner, clustering factors that have been described as influencing influenza vaccination uptake in the selected publications according to social health determinants. Emerging evidence demonstrates that age, gender, healthcare utilization or accessibility, education, income/socioeconomic status and types of chronic diseases are among the most relevant determinants for VH.

Evidence-based decision-making is extremely important to support decision makers and policymakers across the globe, to tackle VH successfully: indeed, multiple approaches and interventions will require collaboration between government, public health institutions and healthcare workers to take appropriate actions are tailored to a local context [60].

This scope review updates the latest information on health determinants impacting vaccination uptake for seasonal influenza among older citizens. Authors made use of the framework of health determinants as introduced by WHO in 2010 [2] to stratify factors associated with VH for seasonal influenza vaccination in older people. As may be seen from this article many publications build evidence for factors associated with reduced vaccination uptake at the micro-level of public health systems. Factors such as age [9–12, 14, 15, 17–22, 25–31, 33, 35–42, 47–49, 51], gender [9–11, 14–22, 25–31, 33–35, 37–42, 47, 49], types of chronic diseases [9–11, 14, 16, 19, 21, 25, 30, 31, 35, 37–39, 42, 49], but also income/socioeconomic status [11, 14, 15, 17, 19–22, 25, 27, 28, 30, 34, 39, 40, 42, 49] and level of education [11, 14–22, 25, 27–30, 34, 39, 41, 42] seem to have a strong impact on vaccination uptake also among older people. This implies a selection of persona, who need to be explicitly targeted by professionals to increase uptake for seasonal influenza vaccination. Patients suffering from chronic diseases such as diabetes or cancer with or without functional deficits as disability [11, 17, 34, 49, 51], with social deprivation [50] or lower economic status [15, 20, 28, 39, 40]. Given the high total numbers of people included in the studies selected during the search process for this article, a high degree of confidence and reliability of the presented content may be assumed.

Beyond those factors, authors were able to find studies further contributing to a deeper understanding of individual barriers to seasonal influenza vaccination uptake: self-reported reasons or attitudes, such as “no time” [20, 23, 42], “forgot” [23], allergic reactions [20] or “didn’t want it” [20, 35] allow a detailed understanding on how to approach older groups of patients in public health to increase vaccination uptake (VU) for seasonal influenza. There is an urgent need now to develop care plans to target VU as past experiences with the 1918 and 1957 influenza pandemics point to the possibility of a resurgence. Given that both SARS-CoV-2 and influenza share similar early symptoms of illness, influenza vaccination status provides an additional mechanism to help distinguish potential infections that could be responsible for a patient’s symptoms.

Additional factors influencing influenza vaccination uptake by older citizens found in this analysis range from influence of family/friends [22, 26, 32, 42] to household arrangements/children [10, 18, 19, 21, 22, 28, 30, 31, 33, 37, 38, 42, 47, 48], previous vaccinations [9, 15, 17–20, 22, 29, 30, 32, 35, 38, 42, 51] and other health parameter [9, 15–21, 25, 27, 28, 31, 35, 39]. This implies that the psychosocial environment, like in children, impacts VU. This is important to notice as only a few models have been proposed, on how strategies to overcome vaccination hesitancy may be framed taking into account the various factors that affect it. Existing validated measures assessing VH focus primarily on confidence in vaccines and the system that delivers them. However, empirical and theoretical work has stated that complacency (not perceiving diseases as high risk), constraints (structural and psychological barriers), calculation (engagement in extensive information searching), and aspects pertaining to collective responsibility (willingness to protect others) also play a role in explaining attitudes towards vaccination play a role. This framework has become known as the 5C model, which builds on and extends the previous general (e.g., health belief model [61]) and vaccine-specific (e.g., 3C model [59]; 5A model [62]) health models/theories. It provides a novel tool to monitor the psychological antecedents of vaccination and facilitates diagnosis, intervention design, and evaluation [63]. Given the gaps detected in our scope review for comprehensive interventions to overcome VH for seasonal influenza, it becomes clear there is an urgent need to implement these models and evaluate their success on a public health level also with other citizens.

Only 23 articles published recently and presented in this review focus on healthcare utilization or accessibility. These also include factors on attitudes and behaviour of physician providing care [24, 40, 49], recommendations released by governmental bodies [13, 33, 51], level of care [17, 38, 48]. However, evidence presented for those domains impacting vaccination uptake by older citizens is rather scarce. It will be important for Governments to encourage as many people as possible to get vaccinated against influenza so as to reduce the burden on health systems. Information on policies or global strategies and how care providers can support the uptake and awareness, however, is weak, at least in the results found in this scope review. Prior factors supporting VU are the dissemination of knowledge; and broad coverage with vaccines, also offering pneumococcal vaccination for older citizens. Mo et al. demonstrated that participation in community activities helped to disseminate the information on the urgency for seasonal influenza vaccination, especially among older women [33]. This information is generally in line with recommendations to support active and healthy ageing released by WHO [64] and also reflects work collectively supported by partners gathered in the European Innovation Partnership on Active and Healthy Ageing (EIP on AHA) [65]. Increased social inclusion and participation lead to more enduring networks of family, friends and carers and greater involvement in social life, all contribute to reduce loneliness, depression and other mental health issues, and are associated with generally improved health.

The information collected within the current review is of pivotal relevance, especially during times of the SARS-CoV-2 pandemic. As shown earlier, one of the key sites for the social exclusion of older people in healthcare [66], opens the need for new communication strategies to disseminate information on the importance of seasonal influenza vaccination, especially for older and isolated population groups. The role of General Practitioners (GPs) is not yet well defined in this context in many European countries. Very often GPs claim a lack of time and capacity to address all their patients, especially by home visits [67]. This raises the demand for low-level access to information delivered by other professions involved in the care process or using relatives, informal caregivers and NGOs as proxies to offer seasonal vaccination and to inform older citizens. The work presented here provides evidence for a public health intervention in frail older people in Brazil delivered by an informal network [21]. In this trial, a significantly positive correlation between social insertion and VU of seasonal influenza vaccination was achieved either by attending church services or religious activities or participation in community centres or groups exclusively designed for seniors. To the best of the authors’ knowledge, this is a single study reporting on this important aspect of social inclusion and vaccination uptake. Given the impact of the topic, however, it is clear that interventions planned in the short term for the incoming autumn/winter season to improve VU among older citizens should be accompanied by a scientific evaluation to allow future learning also for other countries and systems.

Our review faces some shortcomings. Many of the studies included, according to the search strategy, used self-reported information of participants, especially about vaccination uptake, that may be biased. Furthermore, no restriction on the health status of the target group or setting may have a distorting effect on results (e.g. it may be in the inherent nature of people at risk to be vaccinated regularly, other than healthy adults who may not think about preventive measures against infectious diseases—“invisible danger”): the studies included in this review report include data gathered across the globe and many continents. This may primarily be seen as a strength of the work, however, this means “heterogeneity of health systems”, settings and target groups, may lead to diversified results.

Given the high number of persons included in this review (up to a total of 19.604.711 participants), the authors believe that the results presented and reflected in the discussion can have an impact on future decision-making for health care providers. The clear personas profile reflected here, and the individual needs of older people may allow interventions to be tailored, despite the limitation of rare data on evidence for interventions on meso- and macro-level of systems to increase VU among older citizens, especially for seasonal influenza. Given the current urgency due to the SARS-CoV-2 pandemic, this review may help to support older people to maintain their health and independence.

## Conclusions

This scope review presents a range of determinants, especially on individual levels that affect influenza vaccination uptake. Further strategies to increase influenza vaccination uptake and decrease vaccine hesitancy among older adults may build on this evidence. On a system-level, determinants that influence vaccination behaviour among older adults are underexplored up to now and require further research.

## Supplementary Information

Below is the link to the electronic supplementary material.

Supplementary file1 (PDF 132 KB)

Supplementary file2 (PDF 147 KB)

Supplementary file3 (PDF 662 KB)

## Data Availability

Available upon request to the corresponding author.
