# Supplementary material for: The role of health determinants in the influenza vaccination uptake among older adults (65+): a scope review
Source: Aging Clin Exp Res. 2021 Feb 15;33(8):2123–32. doi: 10.1007/s40520-021-01793-3 (PMC7882864; doi:10.1007/s40520-021-01793-3)
Supplement: Supplementary file 1 — Supplementary file1 (PDF 132 KB) [file 40520_2021_1793_MOESM1_ESM.pdf]

The role of health determinants in the influenza vaccination uptake among older adults (65+): a scope review

Aging Clinical and Experimental Research

Regina Roller-Wirnsberger (Corresponding Author, Medical University of Graz, Department of Internal Medicine), Sonja Lindner, Lea Kolosovski, Elisabeth Platzer, Peter Dovjak, Holger Flick, Chariklia Tziraki and Maddalena Illario

Supplementary Table 1: Study Characteristics

No.	Author (Year)	Continent/ Country	Study design and method	Participants and setting	≥65 years subgroup % (sample)	Outcome (Effect estimate)
**1**	Byeon (2018)	Asia/ South Korea	secondary analysis of a cross-sectional study<br>data retrieved from Korea community health survey (KCHS) questionnaire	Survey participants ≥ 50<br>n= 130.610	49%<br>n= 64.134	vaccine uptake (OR)
**2**	Cha (2016)	Asia/ South Korea	cross-sectional study<br>data retrieved from KNHANES Survey	Adults ≥40 years old who underwent pulmonary function testing<br>*n*=3.224	31%<br>*n*=1.013	vaccine uptake (OR)
**3**	Chang (2016)	Asia/ Taiwan	cross-sectional study<br>data retrieved from National Disability Registration System & Health Service Records	Disabled people aged ≥65 years<br>n= 283.172	100%	vaccine uptake (OR)
**4**	Kwon (2016)	Asia/ South Korea	secondary analysis of a cross-sectional study<br>data retrieved from KNHANES IV survey questionnaire	Older adults aged ≥65 years<br>*n*=3.567	100%	vaccine uptake (OR)
**5**	Leung (2017)	Asia/ Hong Kong	randomized controlled trial	Patients (≥65 years old) of two general outpatient clinics<br>*n*=529	100%	vaccine uptake (RR)
**6**	Mo (2015)	Asia/ Hong Kong	cross-sectional study<br>telephone interview	Older adults aged ≥65 years<br>*n*=1.101	100%	vaccine uptake (OR)
**7**	Oh (2015)	Asia/ South Korea	cross-sectional study<br>questionnaire	Adult cancer survivors aged ≥19 years old<br>*n*=35.127	23%<br>*n*=8.172	vaccine uptake (OR)
**8**	Wershof-Schwartz (2013)	Asia/ Israel	cross-sectional study<br>data retrieved from the Maccabi Healthcare Services (MHS)	patients aged ≥65 years which are members of MHS<br>n= 136.944<br>mean age: 74.6 years	100%	vaccine uptake (OR)

**9**	Yu (2014)	Asia/ China	cross-sectional study face-to-face interviews	community-dwelling older adults ≥65 years n= 306	100%	vaccine uptake (OR)
**10**	Dyda (2015)	Australia/ Australia	secondary analysis of a cross-sectional study questionnaire	participants of the cohort study “45 and Up Study” aged ≥ 45 n= 27.036 mean age = 65.6 years	28.8% n= 7.785	vaccine uptake (RR)
**11**	Regan (2017)	Australia/ Australia	randomized controlled trial	high-risk groups *n*=12.354	29% *n*=3.613	vaccine uptake (RR)
**12**	Aguilar (2012)	Europe/ Spain	cross-sectional study data retrieved from vaccination register	Non-institutionalized persons ≥ 65 n= 104.427	100%	vaccine uptake (OR)
**13**	Barbadoro (2016)	Europe/ Italy	secondary analysis of a cross-sectional study data retrieved from “Health and healthcare use in Italy” Survey questionnaire face-to-face interview	Obese adults in Italy, ≥18 years old *n*=10.530 (2005) *n*=11.425 (2013)	63 % *n*=2.086 (2005) 53 % *n*=2.221 (2013)	vaccine uptake (OR)
**14**	Caille-Brillet (2014)	Europe/ France	secondary analysis of a cross-sectional study data retrieved from the CoPanFlu France cohort questionnaire	Participants ≥ 15 years old of the CoPanFlu France cohort n= 1.174	22.9% n= 269	vaccine uptake (OR)
**15**	Domínguez (2016)	Europe/ Spain	cross-sectional study hospital medical records and questionnaire	patients aged ≥65 years hospitalized due to causes unrelated to influenza *n*=1.038	100%	vaccine uptake (OR)
**16**	Ganczak (2017)	Europe/ Poland	cross-sectional study questionnaire	Hospitalized patient ≥65 years old *n*=230	100%	vaccine uptake (OR)
**17**	Giese (2016)	Europe/ Ireland	cross-sectional study telephone interview	adults at-risk and health care workers ≥18 years old *n*=1.770	16% *n*=285	vaccine uptake (PR)
**18**	Godoy (2015)	Europe/ Spain	cross-sectional study regional records and survey	Physician providing patient care for adults ≥65 years *n*=2.535	100%	vaccine uptake (Prevalence)

**19**	Hellfritzsch (2017)	Europe/ Denmark	cross-sectional study questionnaire	Older adults (65–79 years) in a tax-supported healthcare system *n*=4.237	100%	vaccine uptake (PR)
**20**	Martínez-Baz (2012)	Europe/ Spain	cross-sectional study data retrieved from vaccination registry	non-institutionalized persons aged ≥65 years registered and who received seasonal vaccine in 2009/10 season n= 64.245	100%	vaccine uptake (OR)
**21**	Poscia (2016)	Europe/ Italy	Review	frail elderly people (aged ≥65 years)	100%	n.a.
**22**	Shah (2012)	Europe/ England & Wales	cross-sectional study data retrieved from primary care database	continually registered patients aged 65-104 from 312 practices in England & Wales n= 387.568 mean age: 85.5 years	100%	vaccine uptake (RR)
**23**	Spreckelsen (2018)	Europe/ Germany	secondary analysis of a cross-sectional study data retrieved from claims data of GAK Gesundheit (health insurance fund)	nursing home residents aged ≥65 years *n*=42.068	100%	vaccine uptake (OR)
**24**	Verger (2015)	Europe/ France	cohort study data retrieved from NHIF database	patients aged ≥ 18 years treated with OHA for type 2 diabetes n= 110.823	55.4% n= 61.396	vaccine uptake (RR)
**25**	Vukovic (2018)	Europe/ Italy	cohort study data retrieved from Lazio Health and Social Policy Directorate and National Institute for Statistics	elderly subjects aged ≥65 years living in the city of Rome from 2009-2013 n= 554.028-590.603	100%	vaccine uptake (variance)
**26**	Blank (2012)	15 European Countries	cross-sectional study questionnaire	European National Vaccine Industry Groups (NVIGs) (n=16) → relation to vaccine coverage rate among people ≥ 65	100%	vaccine uptake (correlation coefficient Spearman's Rho)
**27**	Banach (2012)	North America/ USA	cross-sectional study data retrieved from MSVD electronic medical record	Patients ≥ 65 receiving routine care from MSVD n= 689 Median age: 86 years	100%	vaccine uptake (OR)

**28**	Black (2017)	North America/ USA	secondary analysis of a cross-sectional study data retrieved from Minimum Data Set (MDS)	U.S. nursing home residents, ≥18 years old *n*=2.446.647 (2005-06) *n*=2.640.219 (2014-15)	86% *n*=2.113.398 (2005-06) 84% *n*=2.215.275 (2014-15)	vaccine uptake (OR)
**29**	Farmanara (2018)	North America/ Canada	secondary analysis of a cross-sectional study data retrieved from Influenza Immunization Coverage Survey telephone interview	Canadian adults ≥18 years old *n*=1.950	26% *n*=514	vaccine uptake (OR)
**30**	Hurley (2018)	North America/ USA	randomized controlled trial data retrieved from CIIS	Adults (19-64 years old) with and without a high-risk condition and adults aged ≥65 years *n*=47.268	11% *n*=5.332	vaccine uptake (OR)
**31**	Kaljee (2017)	North America/ USA	qualitative cross-sectional study focus group discussions	patients at one of three targeted primary care clinics ≥ 65 years n= 48	100%	vaccine uptake
**32**	Khan (2018)	North America/ USA	cross-sectional study data retrieved from BRFSS telephone interview	Older adults aged ≥65 years *n*=13.106.163	100%	vaccine uptake (OR)
**33**	Lu (2014)	North America/ USA	secondary analysis of a cross-sectional study data retrieved from BRFSS survey telephone interview	Non-institutionalized U.S. population aged ≥18 years n= 132.743	44.3% n= 58.814	vaccine uptake (PR)
**34**	Lu (2018)	North America/ USA	secondary analysis of a cross-sectional study data retrieved from National Internet Flu Survey telephone interview	Non-institutionalized U.S. population aged ≥18 years *n*=4.305	20% *n*=1.433	vaccine uptake (PR)
**35**	McIntyre (2014)	North America/ Canada	qualitative cross-sectional study focus group discussions	older adults (aged 67-91) residing in retirement homes or condominium building n= 37 mean age: 82 years	100%	vaccine receipt

**36**	Pereira (2019)	North America/ Canada	cross-sectional study survey	adults aged ≥65 years n= 5.014 mean age: 71.3 years	100%	vaccine uptake (OR)
**37**	Takayama (2012)	North America/ USA	secondary analysis of a cross-sectional study data retrieved from BRFSS telephone interview	Non-institutionalized U.S. population aged ≥ 18 years n= 420.968	31.9% n= 134.101	vaccine uptake (PR)
**38**	Wooten (2012)	North America/ USA	secondary analysis of a cross-sectional study data retrieved from National Adult Immunization Survey (NAIS) telephone interview	adults aged ≥65 years n= 3.821	100%	vaccine uptake (OR)
**39**	Yokum (2018)	North America/ USA	randomized controlled trial	Medicare FFS beneficiaries ≥66 years *n*= 228.000	100%	vaccine uptake (HR)
**40**	Francisco (2015)	South America/ Brazil	secondary analysis of a cross-sectional study data retrieved from FIBRA survey questionnaire	Older adults ≥65 years old *n*=679	100%	vaccine uptake (PR)
**41**	Sato (2015)	South America/ Brazil	cross-sectional study questionnaire	community-dwelling older adults aged ≥60 years *n*=1.341	71% *n*=954	vaccine uptake (PR)
**42**	Doherty (2016)	n.a.	Review	Risk groups	n.a.	n.a.
**43**	Kan (2018)	n.a.	systematic review database search	Older adults aged ≥60 years	n.a.	vaccine uptake
**44**	Thomas (2018)	n.a.	systematic review of RCTs database search	People in the community of high-income countries aged ≥ 60 years n= 1.055.337	n.a.	vaccination uptake (OR)

Supplementary Table 1. Supplementary Table 1 shows study characteristics of 44 studies included into final evaluation of the scope review. As may be seen from the figure data on vaccination uptake were collected in countries on five continents (graphically structured by colour) and within care settings ranging from community to hospital and long-term care. Besides adults, also health and social care workforce were asked about vaccination uptake of influenza vaccination using face to face interviews, questionnaires and surveys, telephone interviews. Analysis from data basis such as the Medicare system were also taken into account. Overall, information was collected from 19.604.711 participants in studies included in the final analysis for this scope review.
