# Supplementary material for: The role of health determinants in the influenza vaccination uptake among older adults (65+): a scope review
Source: Aging Clin Exp Res. 2021 Feb 15;33(8):2123–32. doi: 10.1007/s40520-021-01793-3 (PMC7882864; doi:10.1007/s40520-021-01793-3)
Supplement: Supplementary file 2 — Supplementary file2 (PDF 147 KB) [file 40520_2021_1793_MOESM2_ESM.pdf]

The role of health determinants in the influenza vaccination uptake among older adults (65+): a scope review

Aging Clinical and Experimental Research

Regina Roller-Wirnsberger (Corresponding Author, Medical University of Graz, Department of Internal Medicine), Sonja Lindner, Lea Kolosovski, Elisabeth Platzer, Peter Dovjak, Holger Flick, Chariklia Tziraki and Maddalena Illario

Supplementary Table 2: Analysed factors influencing influenza vaccination uptake in adults > 65 years according to literature search

	Intermediary Determinants																														Structural Determinants											
	Age, sex and constitutional factors						Individual lifestyle factors					Social and community networks				Self-reported barriers/facilitators								Living and working conditions, socioeconomic and healthcare factors															General political, cultural and environmental conditions				
	Age	Gender	Health status	Presence of chronic disease	Type of chronic diseases	Other health parameter	Smoking status	Alcohol intake	Physical activity	Diet	Self-care	Marital status	Household arrangements/Children	Influence of family/friend	Social networks	Experiences	Trust/Belief in efficiency	Attitudes and beliefs	Not needing/Perceived susceptibility	No time	Forgot	Allergy	Didn't want it	Residential area	Healthcare utilization/accessibility	Level of care	Recommendations from Healthcare Professionals	Characteristics GP	Vaccinated before	Price, Free of charge IV	Education level	Knowledge about influenza (vaccine)	Employment	Income/Socioeconomic status	Deprivation	Characteristics GP	Health insurance	Cultural ethnic groups	Recommended by government	Other policy elements	Reminder	Awareness campaigns
Byeon 2018																																										
Cha 2016																																										
Chang 2016																																										
Kwon 2016																																										
Leung 2017																																										
Mo 2015																																										
Oh 2015																																										
Wershof-Schwartz 2013																																										
Yu 2014																																										
Dyda 2015																																										
Regan 2017																																										
Aguilar 2012																																										
Barbadoro 2016																																										
Caille-Brillet 2014																																										
Dominguez 2016																																										
Ganczak 2017																																										
Giese 2016																																										
Godoy 2015																																										

	Intermediary Determinants																														Structural Determinants											
	Age, sex and constitutional factors						Individual lifestyle factors					Social and community networks				Self-reported barriers/facilitators								Living and working conditions, socioeconomic and healthcare factors														General political, cultural and environmental conditions				
	Age	Gender	Health status	Presence of chronic disease	Type of chronic diseases	Other health parameter	Smoking status	Alcohol intake	Physical activity	Diet	Self-care	Marital status	Household arrangements/Children	Influence of family/friend	Social networks	Experiences	Trust/Belief in efficiency	Attitudes and beliefs	Not needing/Perceived susceptibility	No time	Forgot	Allergy	Didn't want it	Residential area	Healthcare utilization/accessibility	Level of care	Recommendations from Healthcare Professionals	Characteristics GP	Vaccinated before	Price, Free of charge IV	Education level	Knowledge about influenza (vaccine)	Employment	Income/Socioeconomic status	Deprivation	Characteristics GP	Health insurance	Cultural ethnic groups	Recommended by government	Other policy elements	Reminder	Awareness campaigns
Hellfritzsch 2017																																										
Martínez-Baz 2012																																										
Poscia 2016																																										
Shah 2012																																										
Spreckelsen 2018																																										
Verger 2015																																										
Vukovic 2018																																										
Blank 2012																																										
Banach 2012																																										
Black 2017																																										
Farmanara 2018																																										
Hurley 2018																																										
Kaljee 2017																																										
Khan 2018																																										
Lu 2014																																										
Lu 2018																																										
McIntyre 2014																																										
Pereira 2019																																										
Takayama 2012																																										
Wooten 2012																																										
Yokum 2018																																										
Francisco 2015																																										
Sato 2015																																										

	Intermediary Determinants																														Structural Determinants											
	Age, sex and constitutional factors						Individual lifestyle factors					Social and community networks				Self-reported barriers/facilitators								Living and working conditions, socioeconomic and healthcare factors														General political, cultural and environmental conditions				
	Age	Gender	Health status	Presence of chronic disease	Type of chronic diseases	Other health parameter	Smoking status	Alcohol intake	Physical activity	Diet	Self-care	Marital status	Household arrangements/Children	Influence of family/friend	Social networks	Experiences	Trust/Belief in efficiency	Attitudes and beliefs	Not needing/Perceived susceptibility	No time	Forgot	Allergy	Didn't want it	Residential area	Healthcare utilization/accessibility	Level of care	Recommendations from Healthcare Professionals	Characteristics GP	Vaccinated before	Price, Free of charge IV	Education level	Knowledge about influenza (vaccine)	Employment	Income/Socioeconomic status	Deprivation	Characteristics GP	Health insurance	Cultural ethnic groups	Recommended by government	Other policy elements	Reminder	Awareness campaigns
Doherty 2016																																										
Kan 2018																																										
Thomas 2018																																										

Supplementary Table 2. Supplementary Table 2 shows the social determinants analysed in the papers included in this scope review on vaccination hesitancy and factors affecting seasonal influenza vaccination uptake among older people. Coloured boxes indicate that the respective determinant has been analysed in the study. Grey boxes point out those determinants that have not been considered within the article concerned. As may be seen from the table there is strong evidence around individual person-related factors that influence vaccination behaviour. Only few publications which were found due to the search strategy applied build evidence for system based-factors or political interventions to improve VU.
