# Supplementary material for: The role of health determinants in the influenza vaccination uptake among older adults (65+): a scope review
Source: Aging Clin Exp Res. 2021 Feb 15;33(8):2123–32. doi: 10.1007/s40520-021-01793-3 (PMC7882864; doi:10.1007/s40520-021-01793-3)
Supplement: Supplementary file 3 — Supplementary file3 (PDF 662 KB) [file 40520_2021_1793_MOESM3_ESM.pdf]

The role of health determinants in the influenza vaccination uptake among older adults (65+): a scope review

Aging Clinical and Experimental Research

Regina Roller-Wirnsberger (Corresponding Author, Medical University of Graz, Department of Internal Medicine), Sonja Lindner, Lea Kolosovski, Elisabeth Platzer, Peter Dovjak, Holger Flick, Chariklia Tziraki and Maddalena Illario

Supplementary Table 3: Determinants associated with increase or decrease of VU

Author	VU increase (+) or decrease (-)	Determinants associated with increase or decrease, respectively
Byeon 2018	**+ men**	• Having a spouse/being married<br>• Former smoker/non-smoker<br>• Walking activities<br>• Health examination<br>• Visit of public health centre<br>• Hypertension, diabetes, cardiovascular diseases
	**+ women**	• Unemployment<br>• Former smoker/non-smoker<br>• Health examinations<br>• Visit of public health centre<br>• Hypertension, diabetes
	**- men**	• Good subjective health status
	**- women**	• Alcohol consumption<br>• Good health status
Cha 2016	+	• Receiving regular health screenings
Chang 2016	+	• Receiving vaccination in previous year<br>• Frequent use of outpatient departments<br>• Undergoing health examinations in previous year
Kwon 2016	+	• Recent history of health screening<br>• Higher age (≥ 70 and ≥ 75)<br>• Self-reported health status as unhealthy
	-	• Smoking<br>• Low physical activity
Leung 2017	+	• Face-to-face patient education and information material
Mo 2015	+	• Female gender<br>• Chronic diseases<br>• Participation in community activities<br>• Knowledge of the fact that vaccine is required every year<br>• Lower perceived side effect<br>• Lower IV price<br>• Recommendations from healthcare providers
Oh 2015	+	• Female gender<br>• Increasing age

		• Having health insurance<br>• Having medical check-up<br>• Co-morbidities<br>• Worse self-related health
Wershof-Schwartz 2013	-	• Female gender<br>• Rural residency<br>• Low socio-economic status<br>• Recent immigration<br>• Being from/Having physician from former Soviet Union
Yu 2014	+	• Perceived susceptibility<br>• Female gender<br>• Multimorbidity<br>• Perceived disease severity<br>• Perceived benefit from current vaccination
	-	• Post-vaccination discomfort
Dyda 2015	+	• Female gender<br>• Higher Body-Mass-Index<br>• Requiring assistance in daily tasks<br>• Reporting chronic diseases
	-	• Smokers<br>• Non-English speaking country of birth
Regan 2017	+	• Text Message Reminder
Aguilar 2012	+	• Major chronic conditions<br>• High level of dependence<br>• More visits to the General Practitioner (GP)<br>• IV in the previous season
	-	• Female gender<br>• Age < 80 or > 94 years<br>• Immigrant status<br>• Previous hospitalization
Barbadoro 2016	+	• Role of local policy in favouring VU
	-	• Younger age (65 -79 years compared to ≥ 80 years)<br>• Medium level education<br>• Absence of chronic diseases<br>• Smoking<br>• No GP contact in the last 12 months
Caille-Brillet 2014	+	• Getting vaccinated in previous 2 seasons
Domínguez 2016	+	• 3 or more GP visits in the previous year<br>• IV in any of the previous 3 seasons<br>• 23-valent pneumococcal polysaccharide vaccination

Ganczak 2017	+	• Younger age (< 70 years)<br>• Living in urban area<br>• Co-morbidities<br>• Vaccinated family members<br>• Being informed about vaccination<br>• Willingness for vaccination next year
Giese 2016	-	• Regarded as not necessary<br>• Not thinking about it<br>• Consider themselves not at risk
	**- Health care workers**	• Regarded as not necessary<br>• Rarely getting influenza disease<br>• Consider themselves not at risk
Godoy 2015	+	• Physician has been vaccinated
	**+ Physician**	• Worried about infecting patients<br>• Believe in effectiveness<br>• Concerned about getting influenza disease
Hellfritzsch 2017	+	• Higher co-morbidity level<br>• Less likely to never have smoked<br>• Higher prevalence of physical activity<br>• Higher prevalence of major physical limitations<br>• Need for assistance with activities of daily living (ADL)
Martínez-Baz 2012	+	• More physician visits per year
	-	• Female gender<br>• Age (65-69 years or > 95 years)<br>• Hospitalized or diagnosed with any major chronic condition in previous year<br>• Haematological cancer or dementia
Poscia 2016	+	• Communication/Awareness campaigns: System of reminders, recalls, information
Shah 2012	+	• Care home patients with & without dementia<br>• Chronic diseases
	-	• Community-dwelling patients with dementia<br>• Area deprivation
Spreckelsen 2018	+	• Vaccination status before nursing home admission<br>• Region (East-Germany compared to West-Germany)<br>• Number of co-morbidities
Verger 2015	No associations found for age group ≥ 65 years	
Vukovic 2018	-	• Low deprivation index
Blank 2012	+	• Good monitoring systems for VU rates

		• Sending personal letters offering free vaccination<br>• Additional policy elements (e.g. awareness campaigns)
Banach 2012	+	• Dementia
	-	• Female gender<br>• Black race<br>• Living alone
Black 2017	+	• Increasing age (≥ 85 years compared to 75-84 years and 65-74 years)<br>• Female gender<br>• Chronic medical conditions associated with higher risk for influenza-related complications
	-	• Race/Ethnicity: Non-hispanic blacks and Hispanic
Farmanara 2018	-	• Younger age (65-70 years compared to > 70 years)<br>• Lower education level<br>• No chronic medical conditions
Hurley 2018	+	• Receipt of any needed vaccine (tetanus, diphtheria, acellular pertussis or pneumococcal besides influenza)<br>• Centralized reminder/recall system
	-	• Prior refusal<br>• Male gender<br>• Older age (≥ 85 years)
Kaljee 2017	Factors affecting VU in general	• Healthcare access and utilization<br>• Communication and information sources<br>• Social networks<br>• Disease experience, knowledge and perceptions<br>• Vaccine experience, knowledge and perceptions
Khan 2018	+	• Disability
	-	• Race/Ethnicity: Non-hispanic blacks and Hispanic
Lu 2014	-	• Member of ethnic minority group<br>• Lower education<br>• Unemployment<br>• Chronic conditions<br>• Last routine check-up > 1 year ago<br>• Absence of personal doctor
Lu 2018	+	• Doctors visit, receiving provider recommendation
McIntyre 2014	+	• Recommendation by, and trust in, health professionals<br>• Believe in effectiveness
	-	• Fear of adverse reactions

		• Believe in resilience
Pereira 2019	+	• High-dose vaccine if free of cost
Takayama 2012	+	• Higher age<br>• Prior diagnoses of chronic conditions (except myocardial infarction and stroke)
	-	• Non-white race<br>• Lower household income<br>• Lacking health care coverage<br>• Smokers<br>• Physical inactivity<br>• Reporting days of poor physical health in past month
Wooten 2012	+	• Believe in effectiveness<br>• Higher education<br>• Doctor's visit during flu season<br>• Believe in personal susceptibility<br>• Little concern of side effects
Yokum 2018	+	• Receipt of single mailed letters
Francisco 2015	+	• Male gender<br>• Slow gait speed<br>• Social involvement
	-	• Higher level of education
Sato 2015	+	• Higher age (≥ 80 years compared to 70-79 years)
Doherty 2016	-	• Negative attitudes and beliefs regarding vaccination<br>• Failure of health care provider to recommend vaccination<br>• Lack of knowledge of vaccine safety and effectiveness<br>• Perceived susceptibility<br>• Lack of awareness of national recommendations
Kan 2018	Factors affecting VU in general	• Demographic factors<br>– Age<br>– Sex<br>– Living with others<br>• Health promotion factors<br>– Health status and self-perceived health status<br>– Health habits and medical service use<br>• Knowledge/information and its sources<br>• Health behaviour factors

		– Threat perception<br>– Perceived barriers<br>– Cues to action<br>– Behavioural beliefs<br>– Subjective norms<br>– Past behaviour
Thomas 2018	+	• Reminder/recall by letter plus leaflet or postcard<br>• Patient outreach by retired teachers<br>• Invitations by clinic receptionists<br>• Patient education by nurses/pharmacists<br>• Patient counselling by medical students<br>• Patient vaccination by nurses<br>• Multiple recall questionnaires<br>• Payments to physician<br>• Physician reminders<br>• Posters in clinics<br>• Chart reviews/benchmarking

Supplementary Table 3. Supplementary Table 3 illustrates the determinants associated with increase or decrease of VU for each article analysed, respectively. Green rows equipped with a plus sign indicate VU increase, red rows with a minus sign display determinants decreasing VU. Some studies did not clearly figure out results of VU increase or decrease but present factors affecting VU in general.
